# Study of the Biological Developmental Characteristics of the Eye in Children After Laser Surgery for the Treatment of Retinopathy of Prematurity

**DOI:** 10.3389/fmed.2021.783552

**Published:** 2022-01-25

**Authors:** Xianlu Zeng, Miaohong Chen, Lei Zheng, Ruyin Tian, Yi Chen, Honghui He, Jian Zeng, Jicang He, Guoming Zhang

**Affiliations:** ^1^Shenzhen Key Laboratory of Ophthalmology, Shenzhen Eye Hospital, Affiliated Shenzhen Eye Hospital of Jinan University, Shenzhen University School of Medicine, Shenzhen, China; ^2^New England College of Optometry, Boston, MA, United States

**Keywords:** retinopathy of prematurity, laser photocoagulation, ocular biology, refractive error, central corneal thickness, lens thickness

## Abstract

**Objective:**

To observe the differences in ocular biology between premature infants who had undergone retinal laser photocoagulation (LP) for retinopathy of prematurity (ROP) and full-term infants and to investigate the relationships between these differences and the development of the refractive state.

**Methods:**

This retrospective, cross-sectional study included 25 children (50 eyes) who had undergone laser treatment for aggressive posterior retinopathy of prematurity (AP-ROP), ROP in zone I requiring treatment, or ROP in zone II requiring treatment in the posterior pole (laser group) and 29 full-term infants (58 eyes) who had not (control group). Basic information, spherical equivalent (SE), and best corrected visual acuity (BCVA) were collected from the two groups. Their mean ages were 7.32 ± 2.85 and 7.34 ± 2.57 years, respectively (*t* = −0.047, *P* = 0.96). Ocular biology data were measured using an IOL Master 700 instrument (Carl Zeiss Meditec AG) and the data were processed using MATLAB (R2016a, Mathworks Inc.). The data markers included central corneal thickness (CCT), anterior and posterior surface corneal curvature radius (CCR), anterior chamber depth (ACD), lens thickness (LT), lens anterior surface curvature radius, lens posterior surface curvature radius, and eye axis length (AL). Optometric data were collected simultaneously and all BCVA values were converted to the logarithm of the minimum angle of resolution (LogMAR) for analysis. The data were statistically analyzed using SPSS software (V.23.0). Independent sample *t*-tests were used for the assessment of ocular biology and refractive indices in both groups of children and Pearson correlation coefficients were used to evaluate the correlations between age, gestational age at birth and ocular biology structural parameters. *P* < 0.05 was considered statistically significant.

**Results:**

Comparisons of ocular biomarkers, refractive status, and BCVA between children in the laser and control groups showed relationships among ocular biomarkers, including the corneal-related parameters of CCT (0.54 ± 0.04 mm and 0.56 ± 0.03 mm, *t* = −2.116, *P* < 0.05), anterior surface CCR (7.53 ± 0.33 mm and 7.84 ± 0.30 mm, *t* = −5.063, *P* < 0.05), posterior surface CCR (6.75 ± 0.34 mm and 7.03 ± 0.24 mm, *t* = −4.864, *P* < 0.05); as well as those related to anterior chamber depth (ACD) were 3.24 ± 0.26 mm and 3.64 ± 0.26 mm, respectively (*t* = −8.065, *P* < 0.05), lens-related parameters (LT) were 3.80 ± 0.19 mm and 3.45 ± 0.16 mm, respectively (*t* = 10.514, *P* < 0.05); anterior lens surface curvature radius were 10.02 ± 0.93 mm and 10.52 ± 0.85 mm, respectively (*t* = −2.962, *P* < 0.05); posterior lens surface curvature radius were 5.55 ± 0.51 mm and 5.80 ± 0.36 mm, respectively (*t* = −2.917, *P* < 0.05), and ocular axis (AL) were 22.60 ± 1.42 mm and 23.45 ± 1.23 mm, respectively (*t* = −3.332, *P* < 0.05). Moreover, comparison of refractive status and BCVA between two groups of children showed an SE of −1.23 ± 3.38 D and −0.07 ± 2.00 D (*t* = −2.206, *P* < 0.05) and LogMAR (BCVA) of 0.12 ± 0.13 and 0.05 ± 0.11 (*t* = 3.070, *P* < 0.05). Analysis of the correlations between age and ocular biomarkers and refractive status of children in the laser and control groups showed correlations between age and ocular biomarkers in the two groups, in which age in the laser group was positively correlated with AL (*r* = 0.625, *P* < 0.05) but not with other biomarkers (*P* > 0.05). Age in the control group was negatively correlated with CCT, ACD, and AL (*r* = 0.303, 0.468, 0.703, *P* < 0.05), as well as with LT (*r* = −0.555, *P* < 0.05), with no correlation with other biomarkers (*P* > 0.05). Analysis of the correlation between age and refractive status of children in both groups showed that the age of children in both laser and control groups was negatively correlated with SE (*r* = −0.528, −0.655, *P* < 0.05) and LogMAR (BCVA) (*r* = −0.538, −0.542, *P* < 0.05). Analysis of the correlations between refractive status and ocular biomarkers in children in the laser and control groups showed that the refractive status in children in the laser group was negatively correlated with AL (*r* = −0.773, *P* < 0.05) but not with other biomarkers in this group (*P* > 0.05). The refractive status of children in the control group was negatively correlated with ACD and AL (*r* = −0.469, −0.734, *P* < 0.05), positively correlated with LT (*r* = 0.364, *P* < 0.05), and was not correlated with other biomarkers in this group (*P* > 0.05). Analysis of the correlations of gestational age at birth with ocular biomarkers and refractive status in children in the laser group showed a positive correlation between gestational age at birth and AL (*r* = 0.435, *P* < 0.05) but no other correlations with the other biomarkers (*P* > 0.05). Moreover, gestational age at birth was negatively correlated with SE (*r* = −0.334, *P* < 0.05) and LogMAR (BCVA) (*r* = −0.307, *P* < 0.05) in children in the laser group.

**Conclusions:**

Compared to full-term infants, the development of CCT, ACD, LT, and AL was relatively delayed after ROP laser surgery, resulting in thin central corneal thickness, steep corneas, shallow anterior chambers, thicker lenses, “rounder” lens morphology, increased refractive power, and short eye axes, leading to the development of myopia. The changes in refractive status were mainly influenced by increased lens thickness. The results of this study showed that the lower the gestational age at birth, the greater the effects on emmetropization in children after ROP, and the more likely the development of myopia.

## Introduction

Retinopathy of prematurity (ROP) is the leading cause of childhood blindness worldwide. In recent years, with the development of neonatal intensive care techniques, the survival rate of preterm and low birth weight infants has been increasing annually, while the incidence of ROP has also increased significantly (1, 2). Retinal LP is currently the gold standard for the treatment of early ROP (3).

Previous studies showed extensive choroidal scarring with retinal atrophy and glial cell proliferation, retinal pigment epithelium loss, choroidal atrophy, and impaired vascularization in the peripheral retina after ROP laser treatment. These histological changes in the fundus may alter and affect the retinal signals required for eye development and/or the scleral reception and response to these signals, delaying or interfering with normal scleral development, resulting in the abnormal development of the anterior segment structures (4). It can be seen that the corneal parameters, anterior chamber depth, and lens thickness in the anterior segment of children after ROP laser also undergo corresponding changes, which are important markers of refractive error (5, 6).

Previous studies have also observed a significant positive correlation between gestational age at birth and anterior chamber depth, lens thickness, and eye axis. Thus, birth gestational age has a large impact on the development of the biological structures of the anterior segment as well as the development of the refractive state. However, the main factors affecting refractive error in children after ROP laser surgery have not been clarified (7, 8).

Therefore, in this study intends to compare the differences in indicators such as the anterior segment, eye axis length and refractive status in children after ROP laser surgery and full-term infants to understand the impact of laser surgery on children's long-term vision, so as to better guide clinical work.

## Approach

### Study Subjects

This retrospective, cross-sectional study included data from a total of 25 children (male: 17; female: 8) aged 3–11 years (mean age 7.32 ± 2.85 years) diagnosed with AP-ROP, requiring treatment in zone I or zone II of the posterior pole, who had completed ROP laser treatment and had complete follow-up data between May 2019 and February 2021 at Shenzhen Eye Hospital. A total of 29 full-term children (male: 16; female: 13), aged 3–11 years (mean age 7.34 ± 2.57 years) were also age-matched in the outpatient clinic (Table 1). The study followed the guidelines of the Declaration of Helsinki, was approved by the hospital ethics committee, and obtained informed parental consent before performing the study. The inclusion criteria were as follows: (1) Children in the laser group who were initially diagnosed with AP-ROP, ROP in zone I requiring treatment, or ROP in zone II of the posterior pole requiring treatment; (2) Children with ROP who received only laser treatment; (3) Children able to undergo continuous follow-up until the lesion had resolved; (4) Children (or their guardians) who agreed to participate and who cooperated with relevant tests and for whom reliable test data were available; (5) Children in whom the same patterns of data collection were used in follow-up visits and for whom the data were complete. The exclusion criteria were as follows: (1) Children with eye diseases other than ROP; (2) Children having received treatment other than laser treatment; (3) Children with a family history of high myopia; (4) Children unable to cooperate in completing tests or in whom poor cooperation had affected the data quality.

**Table 1:** Statistical comparisons of baseline information between the laser and control groups.

**Baseline information**	**Laser group**	**Control group**	***χ2/t***	***P***
Gestational age at birth (W)	28.16 ± 2.15	–	–	–
Birth weight (g)	1,230 ± 585.56	–	–	–
Number of eyes/person	50/25	58/29	–	–
Sex (m/f)	17/8	16/13	0.930	0.34*
Age (years)	7.32 ± 2.85	7.34 ± 2.57	−0.047	0.96

* *X^2^ test; Age was an independent sample t-test*.

## Methods

### ROP Screening Methods

All screened children were administered compound tropicamide eye drops for mydriasis every 5 min for a total of 5 times until the pupil diameter reached 6 mm. Both eyes were then administered one dose of proparacaine hydrochloride eye drops for surface anesthesia before the examination. Wide-angle retinal images were captured using a third-generation wide-angle digital retinal imaging system for children (RetCam 3, Clarity, USA) according to the Chinese ROP screening guidelines (9). Ten images of different orientations (optic disc-centered, macula-centered, temporal, temporal-distal peripheral, superior, superior distal peripheral, nasal, nasal distal peripheral, inferior, and inferior distal peripheral) were taken to ensure that each image had the optimal focal length and appropriate brightness to obtain a clear and complete picture of the fundus tissue structures. All fundus images were reviewed by two retinal specialists for consensus. Binocular indirect ophthalmoscopy with scleral parietal pressure was performed as needed. According to the Early Treatment of Retinopathy of Prematurity (ETROP) criteria, the ROP severity was staged according to the international classification criteria for ROP. Examinations were repeated at specific intervals until the retinal vascularization reached zone III or until the ROP had completely subsided after treatment (10).

### Retinal Laser Photocoagulation

Within 72 h after diagnosis, basal anesthesia was performed using 0.5% procaine (Alcaine, Alcon Laboratories Inc., USA). LP was performed using an 810 nm diode laser (IRIS Medical Oculight SL 810 nm infrared laser; Iris Medical Inc., USA) in conjunction with a 20 D lens. The laser spot was distributed over the entire avascular retina. The laser parameters were as follows: energy: 200–350 mW and a repetitive mode with a 0.2 s exposure interval. The laser treatments were all performed by the same experienced ophthalmologist. Postoperative corticosteroids, antibiotic eye drops, and cycloplegic agents were used for 7 days to reduce postoperative reactions and prevent infection. The first follow-up visit was scheduled on day 7 after treatment and then every 2 weeks or monthly until the ROP had completely subsided, but not necessarily up to the temporal serrated edge (11, 12).

### IOL Master700 Test Method

All subjects were scanned using the same IOL Master 700 ocular biometry device, with a light source wavelength of 1,035–1,077 nm, a scanning depth of 44 mm, a scanning width of 6 mm, a resolution of 22 um, and a scanning speed of 2,000 scan/s. The children were fully communicated with the examiner before the examination and natural blinking was permitted during the examination to avoid factors such as fixation differences and tear film rupture that could affect the results. The measurements were carried out by the same experienced technician (13). Three examinations were performed in each eye to assess the repeatability and reliability of the measured. Images of poor quality and with deviations in the visual axis position were removed; thus, only examination results with the best quality were selected for storage in the device. The following parameters were collected and measured by the IOL Master 700 device: central corneal thickness (CCT), anterior surface corneal curvature radius (CCR), anterior chamber depth (ACD), lens thickness (LT), and eye axis length (AL).

### Optometry and BCVA Examinations

All children in the group were given compound tropicamide drops for mydriasis every 5 min for 4 times until the pupil diameter reached 6 mm, refractive error was measured using a Topcon KR 800 optometer, and retinoscopy was performed 0.5 h later using a band light detector. BCVA was performed using a standard Snellen visual acuity chart, decimal visual acuity was recorded, the optometric data of the two groups of children were separately recorded and finally converted them into logarithmic (LogMAR) visual acuity at the minimum angle of resolution for statistical analysis.

### Image Data Pre-processing

The image data from the IOL Master 700 device were visualized in MATLAB by statistical parametric mapping 12 (SPM12; University College London). The images were extracted from the anterior segment corneal and lens images during segmentation, each segmentation line in the software was converged with the anterior and posterior surfaces of the cornea and lens to match exactly, and the data were saved. The posterior surface CCR, anterior surface curvature radius of the lens, and posterior surface curvature radius of the lens were then extracted (14).

### Statistical Methods

The statistical analysis was performed using SPSS software (V.23.0). Independent-sample *t*-tests were used for measurement data, chi-square tests were used for count data, and Pearson correlation coefficients were used to evaluate the correlations between parameters. *P* < 0.05 was considered statistically significant.

## Results

### Ocular Biomarkers and Refractive Status

There were statistically significant differences in all observation markers between the two groups (*P* < 0.05) (Table 2).

**Table 2:** Comparisons of ocular biology factors and refractive status in the laser and control groups.

**Observation markers**	**Laser group**	**Control group**	***t***	***P***
CCT (mm)	0.54 ± 0.04	0.56 ± 0.03	−2.116	0.04*
Anterior surface CCR (mm)	7.53 ± 0.33	7.84 ± 0.30	−5.063	0.000*
Posterior surface CCR (mm)	6.75 ± 0.34	7.03 ± 0.24	−4.864	0.000*
ACD (mm)	3.24 ± 0.26	3.64 ± 0.26	−8.065	0.000*
LT (mm)	3.80 ± 0.19	3.45 ± 0.16	10.514	0.000*
Curvature radius of the anterior surface of the lens (mm)	10.02 ± 0.93	10.52 ± 0.85	−2.962	0.004*
Curvature radius of the posterior surface of the lens (mm)	5.55 ± 0.51	5.80 ± 0.36	−2.917	0.004*
AL (mm)	22.60 ± 1.42	23.45 ± 1.23	−3.332	0.001*
SE(D)	−1.23 ± 3.38	−0.07 ± 2.00	−2.206	0.03*
LogMAR (BCVA)	0.12 ± 0.13	0.05 ± 0.11	3.070	0.003*

*Two-sample t-tests were performed for all markers*;

* *P < 0.05*.

*CCT, central corneal thickness; CCR, anterior surface corneal curvature radius; ACD, anterior chamber depth; LT, lens thickness; AL, eye axis length; SE, spherical equivalent; BCVA, best-corrected visual acuity; LogMAR, logarithm of the minimal angle of resolution*.

Children who had undergone ROP laser surgery had a thinner central cornea (*t* = −2.116, *P* = 0.04), a steeper cornea (*t* = −5.063, *P* = 0.000; *t* = −4.864, *P* = 0.000), a shallower anterior chamber (*t* = −8.065, *P* = 0.000), and a thicker lens (*t* = 10.514, *P* = 0.000) compared to those in full-term infants. Moreover, the lens was “round” (*t* = −2.962, *P* = 0.004; *t* = −2.917, *P* = 0.004), the eye axis was short (*t* = −3.332, *P* = 0.001), and the visual acuity was poor (*t* = 3.070, *P* = 0.003) (Table 2).

### Correlations of Age With Ocular Biology and Refractive Status

In the laser group, age was positively correlated with AL (*r* = 0.625, *P* = 0.000); negatively correlated with SE (*r* = −0.528, *P* = 0.000) and LogMAR (BCVA) (*r* = −0.538, *P* = 0.000); and not correlated with other markers in this group (*P* > 0.05) (Table 3).

**Table 3:** Correlations of age with eye biology and refractive status in the laser and control groups.

**Observation markers**	**Age of the laser group**	**Age of the control group**
CCT (mm)	*r* = 0.256, *P* = 0.07	*r* = 0.303, *P* = 0.02*
Anterior surface CCR (mm)	*r* = −0.079, *P* = 0.59	*r* = 0.079, *P* = 0.56
Posterior surface CCR (mm)	*r* = −0.109, *P* = 0.45	*r* = −0.068, *P* = 0.61
ACD (mm)	*r* = 0.261, *P* = 0.07	*r* = 0.468, *P* = 0.000*
LT (mm)	*r* = −0.231, *P* = 0.11	*r* = −0.555, *P* = 0.000*
Curvature radius of the anterior surface of the lens (mm)	*r* = 0.234, *P* = 0.10	*r* = 0.063, *P* = 0.64
Curvature radius of the posterior surface of the lens (mm)	*r* = 0.232, *P* = 0.11	*r* = 0.149, *P* = 0.27
AL (mm)	*r =* 0.625, *P* =0.000*	*r =* 0.703, *P* =0.000*
SE (D)	*r =* −0.528, *P* =0.000*	*r* = −0.655, *P* = 0.000*
LogMAR (BCVA)	*r =* −0.538, *P* =0.000*	*r* = −0.542, *P* = 0.000*

*Pearson correlation analysis was performed for all markers*;

* *P < 0.05*.

*CCT, central corneal thickness; CCR, anterior surface corneal curvature radius; ACD, anterior chamber depth; LT, lens thickness; AL, eye axis length; SE, spherical equivalent; BCVA, best-corrected visual acuity; LogMAR, logarithm of the minimal angle of resolution*.

In the control group, age was positively correlated with CCT, ACD, and AL (*r* = 0.303, *P* = 0.02; *r* = 0.468, *P* = 0.000; and *r* = 0.703, *P* = 0.000); negatively correlated with LT (*r* = −0.555, *P* = 0.000) SE (*r* = −0.655, *P* = 0.000), and LogMAR (BCVA) (*r* = −0.542, *P* = 0.000); and not correlated with other markers in this group (*P* > 0.05) (Table 3).

CCT, ACD, and LT changed significantly with age in both groups of children (*P* < 0.05) (Table 3).

### Correlation Between Ocular Biology and Refractive State

In the laser group, refractive status was negatively correlated with AL (*r* = −0.773, *P* = 0.000) but not with other markers (*P* > 0.05) (Table 4).

**Table 4:** Correlations between refractive status and eye biology in the laser and control groups.

**Observation markers**	**SE (laser group)**	**SE (control group)**
CCT (mm)	*r* = −0.231, *P* = 0.11	*r* = −0.087, *P* = 0.52
Anterior surface CCR (mm)	*r* = 0.051, *P* = 0.73	*r* = 0.138, *P* = 0.30
Posterior surface CCR (mm)	*r* = 0.053, *P* = 0.72	*r* = 0.244, *P* = 0.07
ACD (mm)	*r* = 0.101, *P* = 0.49	*r* = −0.469, *P* = 0.000*
LT (mm)	*r* = −0.139, *P* = 0.34	*r* = 0.364, *P* = 0.01*
Curvature radius of the anterior surface of the lens (mm)	*r* = 0.167, *P* = 0.25	*r* = −0.123, *P* = 0.36
Curvature radius of the posterior surface of the lens (mm)	*r* = −0.034, *P* = 0.82	*r* = 0.073, *P* = 0.59
AL (mm)	*r* = −0.773, *P* = 0.000*	*r* = −0.734, *P* = 0.000*

*Pearson correlation analysis was performed for all markers*;

* *P < 0.05*.

*CCT, central corneal thickness; CCR, anterior surface corneal curvature radius; ACD, anterior chamber depth; LT, lens thickness; AL, eye axis length*.

In the control group, refractive status was negatively correlated with ACD and AL (*r* = −0.469, *P* = 0.000; *r* = −0.734, *P* = 0.000), positively correlated with LT (*r* = 0.364, *P* = 0.01), and not correlated with other indices in this group (*P* > 0.05) (Table 4).

In full-term infants, the refractive state of the eye tended to undergo emmetropization, AL growth was accompanied by anterior chamber deepening and lens thinning (*P* < 0.05). In the laser group, the ocular biomarkers were not significantly altered (*P* < 0.05). Hence, the change in refractive state in children after ROP laser surgery was mainly related to the non-significant changes in ACD and LT (Table 4).

### Correlations of Gestational Age at Birth With Ocular Biology and Refractive Status in the Laser Group

In the laser group, gestational age at birth was positively correlated with AL (*t* = 0.435, *P* = 0.002), negatively correlated with SE (*t* = −0.334, *P* = 0.02) and LogMAR (BCVA) (*t* = −0.307, *P* = 0.03), and not correlated with other markers (*P* > 0.05) (Table 5).

**Table 5:** Correlations of gestational age at birth with eye biology and refractive status in the laser group.

**Observation markers**	**Gestational age at birth**
CCT (mm)	*r* = 0.263, *P* = 0.07
Anterior surface CCR (mm)	*r* = 0.032, *P* = 0.83
Posterior surface CCR (mm)	*r* = −0.072, *P* = 0.62
ACD (mm)	*r* = 0.161, *P* = 0.26
LT (mm)	*r* = −0.168, *P* = 0.24
Curvature radius of the anterior surface of the lens (mm)	*r* = 0.086, *P* = 0.55
Curvature radius of the posterior surface of the lens (mm)	*r* = 0.279, *P* =0.05
AL (mm)	*r* = 0.435, *P* = 0.002*
SE (D)	*r* = −0.334, *P* = 0.02*
LogMAR (BCVA)	*r* = −0.307, *P* = 0.03*

*Pearson correlation analysis was performed for all markers*;

* *P < 0.05*.

*CCT, central corneal thickness; CCR, anterior surface corneal curvature radius; ACD, anterior chamber depth; LT, lens thickness; AL, eye axis length; SE, spherical equivalent; BCVA, best-corrected visual acuity; LogMAR, logarithm of the minimal angle of resolution*.

## Discussion

ROP remains the leading cause of visual impairment in children globally. This condition can be prevented with early screening and interventions to avoid visual impairment and ocular atrophy (15). With improved neonatal intensive care techniques, more preterm infants with younger gestational age and lower weight are surviving, resulting in a high prevalence of ROP in many developing countries (16, 17). LP is based on the principle of ablating the avascular zone of the peripheral retina to reduce VEGF overproduction and, thus, induce regression of neovascularization. This procedure is currently the gold standard for the treatment of ROP (18, 19).

### Eye Development Patterns in Normal Children

In full-term infants, most of the growth and development of the eye occurs in the first year of life; thus, the period before 1 year of age is critical one the development of the anterior segment of the eye, emmetropization, and visual development. During the development of the human eye, different components of the optics system develop to prevent refractive errors, a functional tendency known as “emmetropization.” The key to emmetropization is a negative correlation between the length of the eye axis and the refractive powers of both the lens and cornea. As the eye axis grows, the refractive state of the cornea and lens changes, with the anterior chamber deepening while corneal curvature and lens thickness begin to decrease and the corneal gradually flattens (20).

Previous studies investigating the refractive status of adolescents have shown that there are no significant differences in the examination results or final refraction value after pupil recovery between pupil dilation with compound tropicamide drops and with atropine ophthalmic ointment (21). In the past, ophthalmic examination after pupil dilation with atropine ophthalmic ointment for definite diagnosis has mainly been advocated to avoid the occurrence of inadequate relaxation effects due to excessive accommodation. However, the use of atropine ophthalmic ointment leads to the maintenance of pupil dilation for a relatively long time, and side effects such as redness and dryness of the skin or mucosa can occur (22). In addition, the children included in the present study were mostly examined over the weekend and had to attend school on the following Monday. Therefore, tropicamide was administered as it is a short-acting ciliary muscle-paralyzing agent with a more rapid onset and shorter duration of action than atropine ophthalmic ointment, which enabled the restoration of normal vision within the day of examination and avoided affecting the school activities of the children on the following day. Studies have shown that there are no significant differences in the results of ophthalmic examination between the use of atropine and tropicamide, indicating that tropicamide eye drops can serve as a substitute in situations where the use of atropine ophthalmic ointment is not appropriate. Therefore, all children included in this study were provided compound tropicamide drops for pupil dilation prior to the examinations.

### Comparisons of Ocular Biomarkers and Refractive Status Between the Two Groups

We observed a thinner central corneal thickness, steeper corneal morphology, shallower anterior chamber, thicker lens, “round” lens morphology, shorter eye axis, and poorer refractive status in children in the laser group compared to those in full-term infants (Table 2). The causes of these changes in the anterior segment structure and refractive status include changes in retinal tissue anatomy after ROP laser surgery affecting signals from the retina and/or the sclera's reception and response to these signals required for eye development; delayed or affected normal development of the sclera, resulting in “abnormal” anterior segment structure development; and effects on emmetropization of the eye, leading to abnormalities in the development of the anterior segment tissue (10, 11, 23). Laser-treated eyes show a thinner and steeper cornea, which is contrary to the developmental trend of progressively flatter corneal morphology during emmetropization. LP affects the emmetropization process of the postoperative eye by hindering the corneal flattening process. The corneas of children after ROP laser surgery are also steeper than those of full-term infants; these children also show increased refractive error and a higher risk of myopia. Fielder et al. (24) reported that preterm infants had a “thermal defect” of 1.0 to 2.0°C that was never compensated for postnatally compared to full-term infants. Moreover, reduced relative ocular temperature after birth decreases the likelihood of ocular growth in preterm infants, leading to reduced flattening of the corneal geometry during emmetropization, resulting in a steeper corneal shape in myopia. This hypothesis is supported by animal studies in which changes in the “thermal gradient” of the ocular surface were a contributing factor to myopia (25). Thus, steep corneal morphology is related to both prematurity itself and laser treatment, with a combination of contributing factors. Steeper corneas enhance the traction on the central cornea, resulting in a thinner central corneal thickness compared to those in full-term infants.

Wu et al. (26) reported disrupted tissue in the avascular zone of the peripheral retina and blocked local growth signal from the peripheral retina after laser treatment, resulting in altered ciliary or lens development with a thickened lens. We also observed changes in anterior chamber depth and lens, with a thickened lens and a shallow anterior chamber. We speculate that, due to the increased lens thickness, the anterior surface of the lens moved forward, causing the anterior chamber to become shallow, a finding consistent with those of previous related studies (5).

After ROP laser treatment, eye axis development is shorter than that in full-term infants due to extensive peripheral retinal destruction and glial cell proliferation, which alter eye growth and development and affect emmetropization; thus, the process of eye axis growth is hindered (4).

In normal eye development, the refractive state tends to be emmetropic at 9 years of age. The results of our study indicated that ROP laser treatment affected the normal development of the anterior segment structures in children, resulting in steeper corneal morphology, a “rounded” lens, increased refractive power, and a higher incidence of myopia in postoperative children.

### Correlation of Age With Ocular Biomarkers and Refractive Status Between the Two Groups

We observed that the changes in central corneal thickness, anterior chamber depth, and lens thickness in children in the laser group did not change significantly with age compared to full-term infants (Table 3), indicating damage to the tissue of the avascular zone of the retina in the peripheral part of children after ROP laser surgery and blocking of the local growth signal from the peripheral part of the retina, which affected the normal cornea and lens development. These abnormalities in the development of the anterior segment of the eye lead to changes in the refractive power postoperatively, resulting in poorer visual acuity.

We observed a positive correlation between age and eye axis and a negative correlation with refractive status in both the laser and control groups in the present study (Table 3), indicating that the eye axis gradually increases with age and that the BCVA improved gradually in both groups of children, consistent with the natural patterns of eye axis and visual acuity development during postnatal emmetropization. comparisons of the differences in eye axis and BCVA between the two groups showed shorter eye axis and poorer BCVA after ROP laser treatment compared to those in full-term infants. We could not compare the differences in these factors between these two groups of children as they developed over a longer time. Thus, additional studies with larger sample sizes and longer follow-up periods are needed to verify our findings.

### Correlations Between Ocular Biomarkers and Refractive Status in the Two Groups

In this study, the refractive status of full-term infants was negatively correlated with AL and ACD and positively correlated with LT, while the refractive status of children after ROP laser treatment was negatively correlated with AL and not with other biomarkers (Table 4). During emmetropization, the eye axis grows gradually. Following ROP laser treatment in children, ACD and LT are not as significantly developed compared to those in full-term children, which leads to changes in refractive status in children after ROP laser treatment. Fieß et al. (27) observed little correlation between steep corneal morphology and refractive error. Presumably, alterations in lens development play a more important role in changes in refractive development. Garcia-Valenzuela and Kaufman (28) also concluded that children were more prone to refractive error after ROP laser surgery and that changes in refractive status were mainly influenced by changes in the lens. The change in anterior chamber depth is greatly influenced by lens thickness and morphology, which has a greater impact on the refractive status. The results of the present study confirm that the development of the lens in children after ROP laser is blocked, the lens is thicker, and the refractive capacity of the lens is enhanced, which make these children more susceptible to myopia.

### Correlation of Gestational Age at Birth With Ocular Biomarkers and Refractive Status in Children in the Laser Group

The results of the present study analyzing the correlation between gestational age at birth and biological development of the eye in children in the laser group showed that gestational age was positively correlated with the eye axis and negatively correlated with LogMAR (BCVA), while there were no correlations with other markers (Table 5). Thus, the younger the gestational age at birth, the slower the development of the eye axis in preterm children, the shorter the eye axis, and the poorer visual acuity development compared to those in full-term infants. Ozdemir et al. (8) reported a lower gestational age at birth was associated with poorer visual acuity development in preterm children. Their analysis of gestational age at birth, and ocular biological parameters showed that gestational age at birth was positively correlated with anterior chamber depth, lens thickness, and ocular axis length, with a higher correlation with the eye axis length. These previous findings are not entirely consistent with those of the present study. One possible reason for discordance may be the short follow-up period of the children in this study. Therefore, additional studies with a larger population are required to verify these findings.

### Study Limitations

This retrospective study included no pre-term infants without ROP or children with ROP degenerative. Hence, we cannot rule out whether the change in ocular biology and refractive status was entirely due to prematurity itself or to laser photocoagulation. Further confirmation is needed by increasing the subgroup types and sample sizes in the follow-up cases. The severity of the condition on ROP treatment and laser spots that were not completely identical may have resulted in some bias in data analysis. The follow-up periods for both groups of children were short and only one set of cross-sectional phase comparison data was observed, In future studies, the follow-up time will be increased, and the longitudinal follow-up study will be increased.

## Conclusion

### Children Were More Prone to Myopia After ROP Laser Surgery

Compared to full-term infants, pre-mature children who underwent ROP laser surgery showed delayed development of the biological structure of the eye, a steeper cornea, a shallow anterior chamber, a thicker lens, and a shorter eye axis, leading to a greater risk of myopia in postoperatively.

### Myopia Occurred in Children After ROP Laser Surgery Mainly Due to Increased Lens Thickness, for Which Gestational Age at Birth Is Also an Important Cause

The results of this study showed that the occurrence of myopia in children after ROP laser surgery was mainly caused by lens increased thickness. The lower the birth gestational age of the child who underwent post-ROP laser surgery, the more the emmetropization of the eye was affected and the more likely it was to lead to the development of myopia.

## Data Availability Statement

The raw data supporting the conclusions of this article will be made available by the authors, without undue reservation.

## Ethics Statement

The studies involving human participants were reviewed and approved by Medical Ethics Committee of Shenzhen Eye Hospital. Written informed consent to participate in this study was provided by the participants' legal guardian/next of kin.

## Author Contributions

XZ, RT, YC, HH, and GZ: performing the screening and diagnosis of ROP. XZ, HH, and MC: collection and assembly of data. XZ, LZ, JZ, JH, and GZ: data analysis and interpretation. All authors contributed to the study conception and design, manuscript writing, and final approval of manuscript.

## Funding

This work was supported by the Construction Fund of Medical Key Disciplines of Shenzhen (No. SZXK038), Guangdong Provincial Key High-level Clinical Specialty (Supporting Construction Funds of Shenzhen) (No. SZGSP014), Shenzhen-Hong Kong Joint Funded Projects (Category A), and the Project of Discipline Layout (Item No: JCYJ20170817112542555).

## Conflict of Interest

The authors declare that the research was conducted in the absence of any commercial or financial relationships that could be construed as a potential conflict of interest.

## Publisher's Note

All claims expressed in this article are solely those of the authors and do not necessarily represent those of their affiliated organizations, or those of the publisher, the editors and the reviewers. Any product that may be evaluated in this article, or claim that may be made by its manufacturer, is not guaranteed or endorsed by the publisher.
